# Supplementary material for: Autonomic Phenotypes in Chronic Fatigue Syndrome (CFS) Are Associated with Illness Severity: A Cluster Analysis
Source: J Clin Med. 2020 Aug 5;9(8):2531. doi: 10.3390/jcm9082531 (PMC7464864; doi:10.3390/jcm9082531)
Supplement: Supplementary file 1 [file jcm-09-02531-s001.zip › supplementary_files/Supplementary file 1_5.08-scalone.pdf]

Supplementary file 1: Mean results for all individual questions of Chalder Fatigue Scale and mean results of fatigue scales (Fatigue Severity Scale and Fatigue Impact Scale), anxiety and depression for all participants.

ID	CFQ	FSS	FIS	HADS_1	HADS_2	BDI	GROUP
**cfs_001**	29	45	118	8	12	15	sympathetic with dysautonomia
**cfs_002**	27	56	92	10	6	12	sympathetic with dysautonomia
**cfs_003**	28	54	121	10	10	22	sympathetic with dysautonomia
**cfs_004**	29	58	120	8	9	17	parasympathetic
**cfs_005**	22	55	88	13	7	16	parasympathetic
**cfs_006**	16	42	57	10	6	11	balance
**cfs_007**	28	42	91	15	11	20	parasympathetic
**cfs_008**	20	42	45	4	5	9	sympathetic
**cfs_009**	23	48	85	13	12	12	balance
**cfs_010**	21	39	76	13	8	17	balance
**cfs_011**	29	51	112	15	13	26	sympathetic with dysautonomia
**cfs_012**	21	26	65	7	4	5	balance
**cfs_013**	25	54	81	9	11	22	sympathetic with dysautonomia
**cfs_014**	19	45	67	7	5	10	sympathetic
**cfs_015**	26	38	61	9	6	15	balance
**cfs_016**	20	33	93	6	4	10	balance
**cfs_017**	18	30	69	11	14	16	balance
**cfs_018**	28	48	98			43	sympathetic with dysautonomia
**cfs_019**	21	34	60	7	7	8	balance
**cfs_020**	30	63	134	9	11	18	parasympathetic
**cfs_021**	22	47	39	4	7	10	balance
**cfs_022**	27	42	58	5	4	12	balance
**cfs_023**	30	61	134	14	10	31	sympathetic with dysautonomia
**cfs_024**	23	42	87	13	9	13	balance
**cfs_025**	30	53	111	12	10	17	sympathetic with dysautonomia
**cfs_026**	27	60	100	9	12	27	parasympathetic
**cfs_027**	29	43	96	12	6	16	balance
**cfs_028**	28	46	72	10	7	16	sympathetic with dysautonomia
**cfs_029**	30	54	106	9	11	29	parasympathetic
**cfs_030**	29	63	140	16	11	28	parasympathetic
**cfs_031**	28	52	100	9	7	15	sympathetic with dysautonomia
**cfs_032**	27	47	105	6	2	16	balance
**cfs_033**	23	44	87	10	7	23	parasympathetic
**cfs_034**	26	54	74	6	9	9	parasympathetic
**cfs_035**	24	47	108	9	12	27	parasympathetic
**cfs_036**	31	46	99	13	6	22	parasympathetic
**cfs_037**	20	35	26	11	10	16	sympathetic
**cfs_038**	27	45	80	12	6	18	sympathetic with dysautonomia
**cfs_039**	27	54	92	9	6	9	parasympathetic
**cfs_040**	22	42	63	8	5	5	balance
**cfs_041**	32	63	148	13	17	32	parasympathetic
**cfs_042**	24	54	78	6	5	14	balance
**cfs_043**	25	51	87	4	5	9	balance
**cfs_044**	27	58	96	14	7	20	sympathetic with dysautonomia
**cfs_045**	28	51	99	11	13	20	sympathetic with dysautonomia
**cfs_046**	21	42	93	12	6	14	sympathetic with dysautonomia
**cfs_047**	25	59	111	9	11	26	sympathetic with dysautonomia
**cfs_048**	21	38	57	8	6	15	balance

**cfs_049**	22	48	83	4	12	20	balance
**cfs_050**	23	53	107	9	5	4	sympathetic with dysautonomia
**cfs_051**	26	57	98	8	10	19	parasympathetic
**cfs_052**	21	44	93	11	7	13	sympathetic with dysautonomia
**cfs_053**	27	60	108	9	11	15	parasympathetic
**cfs_054**	31	39	80	12	8	13	balance
**cfs_055**	26	58	120	14	10	28	parasympathetic
**cfs_056**	29	57	110	14	11	24	sympathetic with dysautonomia
**cfs_057**	21	55	121	14	13	34	sympathetic with dysautonomia
**cfs_058**	26	47	110	8	14	29	sympathetic with dysautonomia
**cfs_059**	32	63	153	10	14	19	sympathetic with dysautonomia
**cfs_060**	29	41	94	4	7	8	sympathetic with dysautonomia
**cfs_061**	25	48	87	11	9	29	sympathetic with dysautonomia
**cfs_062**	21	58	91	14	11	16	parasympathetic
**cfs_063**	20	37	67	5	6	15	sympathetic with dysautonomia
**cfs_064**	28	62	114	6	8	28	sympathetic with dysautonomia
**cfs_065**	24	49	87	10	7	11	balance
**cfs_066**	14	29	18	9	7	15	balance
**cfs_067**	12	28	15	3	4	6	sympathetic
**cfs_068**	22	15	16	12	10	8	balance
**cfs_069**	22	54	61	15	2	17	sympathetic with dysautonomia
**cfs_070**	22	47	67	8	9	15	balance
**cfs_071**	18	45	80	11	10	25	parasympathetic
**cfs_072**	21	15	21	1	6	9	balance
**cfs_073**	22	51	74	6	7	3	balance
**cfs_074**	18	18	16	8	3	14	balance
**cfs_075**	27	51	66	9	6	14	parasympathetic
**cfs_076**	19	51	47	10	7	14	balance
**cfs_077**	21	48	22	11	5	13	balance
**cfs_078**	17	41	49	7	2	12	balance
**cfs_079**	22	54	72	15	6	27	parasympathetic
**cfs_080**	22	49	46	14	7	11	sympathetic with dysautonomia
**cfs_081**	19	41	21	6	2	2	balance
**cfs_082**	26	48	61	14	9	21	parasympathetic
**cfs_083**	26	55	81	13	5	13	sympathetic with dysautonomia
**cfs_084**	19	46	18	14	4	11	sympathetic
**cfs_085**	21	46	60	5	2	2	balance
**cfs_086**	21	49	12	8	8	6	balance
**cfs_087**	19	56	44	6	2	9	sympathetic with dysautonomia
**cfs_088**	21	16	67	6	2	4	balance
**cfs_089**	19	46	54	5	5		balance
**cfs_090**				9	10	11	balance
**cfs_091**	22	48	27	7	11	7	balance
**cfs_092**	21	47	72	10	10	16	balance
**cfs_093**	21	46	58	5	6	7	sympathetic with dysautonomia
**cfs_094**	11	49	71	7	5	18	sympathetic with dysautonomia
**cfs_095**				6	5	16	balance
**cfs_096**	28	54	92	4	11	11	sympathetic with dysautonomia
**cfs_097**	26	46	69	7	6	9	balance
**cfs_098**	26	52	91	10	12	24	sympathetic with dysautonomia

**cfs_099**	33	54	96	16	11	17	sympathetic with dysautonomia
**cfs_100**	17	48	49	1	4	6	balance
**cfs_101**	23	49	86	11	15	21	sympathetic with dysautonomia
**cfs_102**	15	42	29	3	3	7	balance

CFQ_QUESTIONS

ID	Do you have problems with tiredness?	Do you need to rest more?	Do you feel sleepy or drowsy?	Do you have problems starting things?	Do you lack energy?	Do you have less strength in your muscles?	Do you feel weak?	Do you have difficulties concentrating?	Do you make slips of the tongue when speaking?	Do you find it more difficult to find the right word?	How is your memory?
**cfs_001**	3	3	3	2	3	2	3	2	2	3	3
**cfs_002**	3	3	2	3	3	3	3	2	2	1	2
**cfs_003**	3	3	3	2	3	2	2	3	2	2	3
**cfs_004**	3	3	3	3	3	2	3	3	2	2	2
**cfs_005**	2	2	2	2	2	2	2	2	2	2	2
**cfs_006**	2	2	2	2	0	2	1	2	2	1	2
**cfs_007**	3	2	2	2	3	2	3	2	2	3	1
**cfs_008**	2	2	3	2	3	0	2	2	2	2	3
**cfs_009**	3	2	3	3	2	2	1	3	1	1	2
**cfs_010**	2	2	2	3	2	1	2	2	2	1	2
**cfs_011**	3	3	3	2	3	2	3	2	3	2	3
**cfs_012**	3	2	3	1	3	2	2	2	1	1	1
**cfs_013**	3	3	2	2	3	2	2	2	2	2	2
**cfs_014**	2	2	1	2	2	2	2	2	1	1	2
**cfs_015**	3	3	3	3	3	1	2	3	2	1	2
**cfs_016**	2	2	2	2	2	2	2	2	1	1	2
**cfs_017**	2	2	2	2	2	0	1	2	2	2	1
**cfs_018**	3	3	3	2	3	3	2	2	2	2	3
**cfs_019**	2	2	2	2	2	2	2	2	2	1	2
**cfs_020**	2	2	2	3	3	3	3	3	3	3	3
**cfs_021**	3	3	2	2	3	3	2	0	2	0	2
**cfs_022**	3	2	3	2	3	3	2	2	3	2	2
**cfs_023**	3	3	3	3	3	3	3	3	2	2	2

**cfs_024**	2	2	2	3	2	2	2	3	1	1	3
**cfs_025**	3	3	3	3	3	3	3	3	3	3	3
**cfs_026**	3	2	3	3	3	3	3	3	0	2	2
**cfs_027**	3	3	2	3	3	3	2	3	2	2	3
**cfs_028**	3	3	3	2	3	3	3	2	2	2	2
**cfs_029**	3	3	3	3	2	3	3	2	3	3	2
**cfs_030**	3	3	3	2	3	3	3	3	2	1	3
**cfs_031**	3	2	2	2	2	3	2	3	3	3	3
**cfs_032**	3	3	0	3	3	1	3	3	3	2	3
**cfs_033**	2	2	2	2	3	3	3	1	2	1	2
**cfs_034**	3	3	3	1	3	3	3	2	1	2	2
**cfs_035**	2	2	2	2	2	1	2	2	3	3	3
**cfs_036**	3	3	3	3	3	3	3	3	3	2	2
**cfs_037**	2	2	2	2	2	2	2	2	1	1	2
**cfs_038**	3	2	3	2	3	3	2	2	3	2	2
**cfs_039**	3	3	3	1	3	3	3	2	2	2	2
**cfs_040**	2	2	2	1	2	1	2	2	3	2	3
**cfs_041**	3	3	3	3	3	3	3	2	3	3	3
**cfs_042**	2	2	3	2	3	2	2	1	2	3	2
**cfs_043**	3	2	3	2	2	2	3	2	1	2	3
**cfs_044**	3	3	3	3	3	3	2	2	2	3	3
**cfs_045**	3	2	3	2	3	3	2	2	3	2	3
**cfs_046**	3	2	2	1	2	2	2	2	1	2	2
**cfs_047**	3	3	3	2	3	2	3	2	1	0	3
**cfs_048**	2	2	2	2	2	2	2	2	2	1	2
**cfs_049**	3	2	3	2	3	2	2	2	0	1	2
**cfs_050**	2	2	2	1	2	2	2	2	3	3	2
**cfs_051**	2	2	2	3	2	3	3	3	3	3	2
**cfs_052**	1	2	2	2	2	1	2	2	2	2	3
**cfs_053**	2	3	3	2	3	3	3	2	2	1	3

**cfs_054**	3	3	3	2	3	3	3	3	3	2	3
**cfs_055**	3	3	3	3	3	2	3	2	2	2	2
**cfs_056**	2	3	2	3	3	2	3	3	2	3	3
**cfs_057**	2	3	2	2	2	2	2	2	2	0	2
**cfs_058**	3	3	3	1	3	1	1	3	2	3	3
**cfs_059**	3	3	3	2	3	3	3	3	3	3	3
**cfs_060**	3	3	3	3	2	2	2	3	3	3	2
**cfs_061**	2	2	3	2	3	2	3	2	2	2	2
**cfs_062**	2	2	2	2	3	3	3	3	1	1	1
**cfs_063**	2	2	3	2	2	2	2	3	1	1	2
**cfs_064**	3	3	3	3	3	2	3	2	2	2	2
**cfs_065**	3	2	3	3	3	3	2	2	1	2	0
**cfs_066**	1	1	2	1	2	0	1	2	1	1	2
**cfs_067**	2	1	0	0	2	2	2	2	0	0	1
**cfs_068**	2	2	3	2	2	3	3	2	1	0	2
**cfs_069**	2	3	2	2	2	1	2	2	2	2	2
**cfs_070**	2	2	2	2	2	2	2	2	2	2	2
**cfs_071**	2	1	2	1	2	1	2	2	1	1	3
**cfs_072**	3	3	3	1	3	2	2	1	1	2	2
**cfs_073**	2	2	3	2	2	2	2	2	2	2	1
**cfs_074**	2	2	2	1	1	1	2	2	1	2	2
**cfs_075**	3	3	3	3	3	1	2	2	2	2	3
**cfs_076**	1	2	2	2	1	2	2	2	2	2	1
**cfs_077**	2	2	2	2	2	3	3	2	1	1	1
**cfs_078**	2	2	1	2	2	2	2	1	1	1	1
**cfs_079**	2	2	2	2	2	1	2	3	2	2	2
**cfs_080**	2	2	2	2	2	2	2	2	2	2	2
**cfs_081**	2	2	2	2	2	1	1	2	2	2	1
**cfs_082**	3	3	3	2	2	2	2	3	2	2	2
**cfs_083**	3	2	3	2	3	3	3	2	1	2	2

**cfs_084**	2	2	2	1	2	1	2	2	2	2	1
**cfs_085**	3	2	2	2	2	2	2	2	2	2	2
**cfs_086**	2	2	2	2	3	2	2	3	1	1	1
**cfs_087**	1	2	2	2	2	1	1	2	2	2	2
**cfs_088**	2	2	2	2	2	2	2	2	2	2	1
**cfs_089**	2	2	2	1	2	1	1	2	2	2	2
**cfs_090**	1	2	2	2	1	1	2	1	1	1	1
**cfs_091**	2	2	3	2	2	1	2	2	2	2	2
**cfs_092**	2	2	2	2	2	2	2	2	2	2	3
**cfs_093**	2	2	2	2	2	1	2	2	2	2	2
**cfs_094**	3	3	3	2	3	3	3	3	3	2	3
**cfs_095**	1	2	2	2	2	2	2	2	2	2	1
**cfs_096**	3	3	3	3	3	3	3	2	2	1	2
**cfs_097**	2	3	3	3	2	2	2	3	2	2	2
**cfs_098**	3	3	2	2	3	3	3	3	2	2	0
**cfs_099**	3	3	3	3	3	3	3	3	3	3	3
**cfs_100**	2	2	1	2	2	2	1	1	1	1	2
**cfs_101**	3	3	2	3	3	2	2	3	3	2	3
**cfs_102**	2	2	2	1	2	1	1	1	1	1	1
