# Supplementary material for: Autonomic Phenotypes in Chronic Fatigue Syndrome (CFS) Are Associated with Illness Severity: A Cluster Analysis
Source: J Clin Med. 2020 Aug 5;9(8):2531. doi: 10.3390/jcm9082531 (PMC7464864; doi:10.3390/jcm9082531)
Supplement: Supplementary file 1 [file jcm-09-02531-s001.zip › supplementary_files/Supplementary file_02_15.07.pdf]

Table S1. Relationship between questions answers in CFQ and arterial stiffness parameters in linear regression analysis.

	PWVaortic			Aixaortic			SBPaortic		
	**Beta**	**p**	**R2**	**Beta**	**p**	**R2**	**Beta**	**p**	**R2**
CFQ_Do you have problems with tiredness?	0.27	0.07	0.60	0.06	0.70	0.60	0.15	0.34	0.60
CFQ_Do you need to rest more?	-0.04	0.77	0.56	-0.13	0.36	0.56	-0.09	0.56	0.56
CFQ_Do you feel sleepy or drowsy?	-0.26	0.03	0.32	-0.18	0.13	0.32	-0.31	0.01	0.31
CFQ_Do you have problems starting things?	-0.19	0.11	0.31	-0.12	0.29	0.31	-0.04	0.75	0.34
CFQ_Do you lack energy?	0.04	0.79	0.53	-0.06	0.66	0.53	-0.15	0.30	0.52
CFQ_Do you have less strength in your muscles?	0.03	0.85	0.48	-0.02	0.90	0.48	0.00	0.98	0.48
CFQ_Do you feel weak?	0.14	0.31	0.50	0.38	0.01	0.50	0.14	0.31	0.50
CFQ_Do you have difficulties concentrating?	-0.01	0.96	0.24	-0.04	0.69	0.24	0.00	0.97	0.24
CFQ_Do you make slips of the tongue when speaking?	-0.17	0.21	0.50	-0.03	0.83	0.50	-0.20	0.15	0.50
CFQ_Do you find it more difficult to find the right word?	0.01	0.92	0.43	0.23	0.07	0.43	0.25	0.06	0.43
CFQ_How is your memory?	-0.12	0.30	0.25	-0.01	0.93	0.25	0.10	0.41	0.25
Total*		0.049	0.19 (0.09)		0.14	0.16 (0.05)		0.24	0.14 (0.03)

**Total denotes results of linear regression model (p value and multiple R2 (adjusted R2)*
